# Prevalence of Temporomandibular Disorders (TMD) in Dental Patients at a Specialized Regional Medical Center in Italy

**DOI:** 10.7759/cureus.60819

**Published:** 2024-05-21

**Authors:** Bachar Reda, Luca Contardo, Gabriele Vidoni, Abbass El-Outa

**Affiliations:** 1 Medical, Surgical and Health Sciences, School of Dentistry, University of Trieste, Trieste, ITA; 2 Research, American University of Beirut, Beirut, LBN; 3 Dentistry & Oral Medicine, Private Practice, Beirut, LBN

**Keywords:** tmd, orofacial pain, epidemiology, diagnosis, chronic pain

## Abstract

Background and aim: This study aimed to evaluate the prevalence of temporomandibular disorders (TMDs) in a sample of general dental patients seeking dental treatments in a northeastern Italian university clinic.

Materials and methods: Records of all patients presented for the first time to the dental division of Maggiore Hospital, Italy, between January 1, 2016, and December 31, 2017, were collected. Patients comprised those presenting to the dental clinics for non-TMD complaints, who, upon general examination, were found to have TMD signs and were referred for TMD evaluation. Data were extracted and analyzed, retrospectively. The prevalence of TMDs, age, gender, signs, and symptoms were evaluated.

Results: Out of the 18,774 patients studied, 284 had signs of TMD. Women predominance was evident (73%), and patients aged 45-50 were the most frequent sub-population within the TMD population. Clicking was the most commonly present symptom (26.8%), and arthralgia was most commonly diagnosed among this sample (30.7%). A considerable number of patients suffered from muscular disease (myalgia and myofascial pain with 10.1% and 20.7% of the patients, respectively). Significant associations were found among those with myofascial pain on the one hand and degenerative disease and disc displacement with reduction, on the other hand. Furthermore, disc displacement with reduction on one side was associated with displacement without reduction on the other side.

Conclusion: A considerable number of patients presenting with dental complaints may have asymptomatic TMDs. This highlights the importance of systematic screening of dental patients for TMDs as part of general assessment.

## Introduction

Temporomandibular disorders (TMDs) correspond to a group of disorders involving the stomatognathic system: the temporomandibular joint (TMJ), masticatory muscles, and the surrounding hard and soft tissues [1]. Patients experiencing such disorder may suffer from one or more symptoms, such as headache or neck pain, ear pain, pressure or tinnitus, and facial and dental pain. Moreover, other symptoms such as functional limitations (e.g., irregular deviations or limited range of movements) are present, as well as joint sounds (clicking, popping, and crepitus) [2]. In addition, patients with TMDs report a negative impact on their daily lives in association with comorbidities and psychosocial defacement [3].

The prevalence of TMDs is highly dependent on the included signs and symptoms and their occurrence in frequency and duration [4]. It has been reported that TMD pain affects approximately 10% of the adult population [5] and is considered to be the most common chronic oro-facial pain condition worldwide. If all symptoms involving TMD are included, the prevalence may increase by up to 50% of the adult population [6].

The prevalence reported in the literature may vary from one study to another. Still, the Research Diagnostic Criteria for Temporomandibular Disorders (RDC/TMD) [7], updated in the DC/TMD in 2014, enabled TMD specialists to standardize their classifications and criteria. This allowed comparing the prevalence of such disorders among studies with different settings. This is an essential step to understanding the different distributions of TMDs [8]. In particular, such instruments could be used to screen the patients in non-specialist settings.

The need for the study on the prevalence of TMDs among general dental patients primarily stems from the underdiagnosis and the potential oversight of these conditions in routine dental care. TMDs, which affect the jaw joint and muscles, can significantly impact an individual's oral health, overall well-being, and quality of life. However, symptoms are often not immediately associated with dental issues. This study highlights the frequency and characteristics of TMDs in a dental patient population, emphasizing the critical need for systematic screening for TMDs during general dental assessments.

Based on this premise, this study aimed to evaluate the prevalence of TMDs in a sample of patients seeking advice at general dentistry in a tertiary-care hospital in Italy (Clinica di Chirurgia Maxillofacciale Odontostomatologica di Trieste). The frequency of signs and a comparison with other studies will also be discussed. The study also seeks to improve awareness among dental professionals, prompting earlier detection, better management, and, ultimately, enhancing patient care outcomes. This approach could lead to more targeted treatments, reducing the burden of TMDs and improving the quality of life for those affected.

## Materials and methods

Clinical records of all patients seeking care for the first time at the Dental Clinic of the Maggiore Hospital, Trieste, Italy, between January 1, 2016, and December 31, 2017, were screened. Data of patients who had signs of TMD according to the DC/TMD were extracted and analyzed retrospectively. The prevalence of TMD, age, gender, signs, and symptoms were described.

Patients under 18 years old and those whose files did not report any specific diagnosis were excluded from the study. All patients provided their informed consent at their initial presentation.

Clinical records showed 18,774 new examinations at the dental clinic over the two years. Two hundred eighty-four patients were referred to the Operative Unit of Gnathology for possible signs or symptoms of temporomandibular disorders. Based on exclusion criteria, data of 179 patients were described.

All new patients were examined for signs of TMDs and were referred to the Gnathology Department by primary care dentists based on routine extra- and intra-oral examination and functional jaw movements. For TMJ examination screening purposes, primary care dentists relied on the guidelines of international academies (i.e., DC/TMD, version: 20 Jan 2014) [9].

Patients were categorized into groups based on the presence of the following signs/symptoms and/or diagnosis: clicking, localized myalgia (defined as sustained palpation with identification of spreading pain but no referral patterns), myofascial pain (defined as sustained palpation with identification of circulating pain but no referral patterns), disc displacement with/without reduction with/without intermittent locking, headache attributed to TMD (headache of any type in the temporal region affected by jaw movement, function or parafunction diagnosed by history), degenerative joint disease, articular disorder, subluxation, fracture, and finally non-TMD group (included patients who presented for night guard without having any TMD).

Data entry and statistical analyses were conducted using IBM SPSS Statistics for Windows (version 24; IBM Corp., Armonk, NY). Descriptive statistics were carried out for all variables. The normality of the distribution of continuous variables was assessed using graphical plots and verified with a one-sample Kolmogorov-Smirnov test. Chi-square tests (Table 1) were done to evaluate differences within each variable and to compare frequencies between categorical variables. The level of significance for the aforementioned tests was set below p=0.05.

**Table 1:** Association testing between different temporomandibular disorders DDwR: disc displacement with reduction; DDw/oR: disc displacement without reduction; DJD: degenerative joint disease

Variables	Test	p
DDwR – Myofascial pain	Fisher’s exact test	0.011
Myofascial pain – DJD	Fisher’s exact test	0.046
DDwR one side, DDw/oR the other side	Chi-square test	0.005

## Results

Common signs and symptoms of the studied 179 patients included myalgia, myofascial pain with/without referral, headache attributed to TMD, arthralgia, disc displacement with/without reduction or with/without intermittent locking, degenerative joint disease, and subluxation.

**Figure 1:**
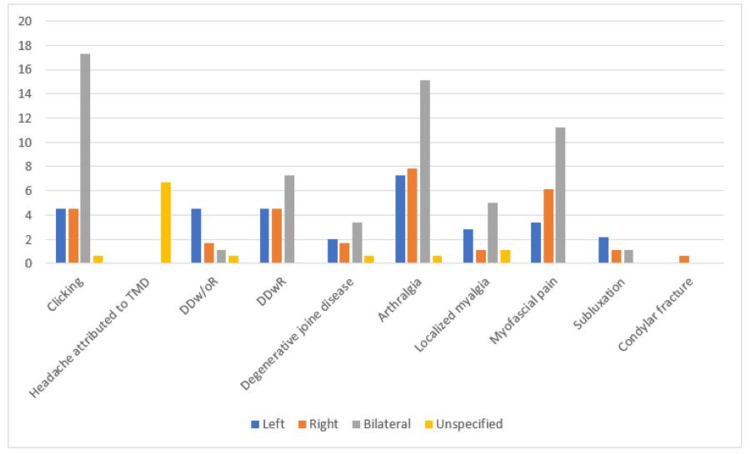
Bar graph showing percentages of prevalent TMD signs and symptoms in the studied sample TMD: temporomandibular disorder; DDw/oR: disk displacement without reduction; DDwR: disk displacement with reduction

Female predominance was evident (73% females), and the male percentage was (27.4%) (Table 1). Age ranged from 18 to 89 years with a mean of 48 years ± 16.015 (Table 2). A quarter of the patients suffered from clicking (26.8%), while the most common disorder was arthralgia (30.7%), followed by myofascial pain (20.7%) (Table 2). Furthermore, myofascial pain was significantly associated with disk displacement with reduction (p=0.011) and degenerative joint disease (p=0.046) (Table 2). Additionally, patients with unilateral disk displacement with reduction were 18.96 times more likely to suffer from disk displacement without reduction on the other side (p<0.001, crude OR=18.96) (Figure 1). Moreover, regression analysis showed that females were 3.26 times more likely to suffer from disc displacement with reduction (DDwR) than males (p=0.041, CI=1.05, 10.14). Additionally, those with myofascial pain had decreased odds of DDwR (aOR=0.095, p=0.024, CI=0.12, 0.73). Additionally, patients with unilateral disk displacement with reduction were 18.96 times more likely to suffer from disk displacement without reduction on the other side (p<0.001, crude OR=18.96) (Figure 1). Moreover, regression analysis showed that females were 3.26 times more likely to suffer from DDwR than males (p=0.041, CI=1.05,10.14). Additionally, those with myofascial pain had decreased odds of DDwR (aOR=0.095, p=0.024, CI=0.12, 0.73).

**Table 2:** Descriptive statistics of the obtained results TMD: temporomandibular disorder; DDw/oR: disk displacement without reduction; DDwR: disk displacement with reduction

				N
		Mean		Standard Deviation
Age		48		
		Count		Percentage
Gender	Female	130		72.6
	Male	49		27.4
Clicking		48		26.8
Diagnosis				
Headache attributed to TMD		12	6.8	
DDw/oR		14	7.8	
DDwR		29	16.2	
Degenerative joint disease		16	8.9	
Arthralgia		55	30.7	
Localized myalgia		18	10.1	
Myofascial pain		37	20.7	
Subluxation		8	4.5	
Condylar fracture		1	0.6	
Non-TMD		61	34.1	

## Discussion

This study aimed to evaluate the prevalence of TMD in dental patients who presented at Maggiore Hospital in the years 2016 and 2017. Out of 18,774 dental patients, 284 (1.5%) were referred to the gnathology department for signs of TMD, of which only 179 (0.96%) fit the inclusion criteria of this study. Different studies evaluating the prevalence of TMD in patients attending general dentistry clinics have reported variable results in the literature. Talaat et al. studied the prevalence of TMD that was observed incidentally in 3,009 patients requesting for routine dental treatments [10]. They reported a prevalence of 10.8%.

Furthermore, they noticed female predisposition with 65.8% and higher TMD prevalence between the ages 25 and 45 years. The latter finding did not agree with our study, which revealed the highest existence between ages 45 and 50, but female predominance was similar (72%). Moreover, the mean age of this study sample (47.75 years) was different than that reported in other studies, such as Manfredini et al. (39.71 years) [11].

AlShaban et al. [12] reported a high prevalence of 41% in patients present with dental complaints. In contrast to other studies, they noticed that males were significantly more affected than females (65%). The highest incidence was recorded in the young age group (19-29 years), with 58% of their studied sample affected with TMD. Concerning symptoms, the striking majority complained of clicking (89%) [12]. In another study, De Oliveira Reis et al. reported a similar prevalence of 53.9% in patients present for dental treatment [13]. Gender differences were significant, with 67.3% female preference and with highest incidence in the age group 41-60 years (52.7%) [13]. Chatzopoulos et al. conducted a similar study on a random sample of 4,204 patients present at university dental clinics and retrospectively analyzed data for TMD symptoms. The most common TMD symptom was clicking (14.8%) [14].

In a recent study, Pantoja et al. reported that degenerative joint disease in patients with TMD ranges from 18% to 84.7%, more than that in our study (8.9%) [15]. Some authors attributed the variability in prevalence rates to inadequate detection of TMDs. Simmons discussed the insufficient training of dentists in diagnosing temporomandibular disorders [16]. Similarly, dentists' distress in diagnosing and managing TMD pain was shown in a study by Yokoyama et al. [17]. Other studies reported similar conclusions, not only with the lack of standardized physical evaluation [18] but also with the lack of psychological assessment [19]. This factor may add to the variation in diagnosing TMDs and lead to lower prevalence.

It is important to mention that our studied sample included 18,774 patients, whereas other studies inspecting the prevalence of signs of TMD were done on a smaller sample size (up to 4,000). Consequently, our study calls for further large-scale studies.

One limitation of our study was the number of patients with missing details excluded from the analysis (105 subjects). In addition, another limitation lies in discrepancies in following the DC/TMD by primary care dentists in the hospital.

## Conclusions

The results of our study suggest that there is under-diagnosis of TMDs. This warrants the call for more emphasis on TMD screening in dental schools, CE programs for dental professionals, and subsequent management and specialist referral.
